# Combined use of DDGP and IMRT has a good effect on extranodal natural killer/T‐cell lymphoma, nasal type

**DOI:** 10.1002/hon.2637

**Published:** 2019-12-01

**Authors:** Yingjuan Zheng, Chunzhao Yang, Tiansong Liang, Daoke Yang, Zhangsuo Liu

**Affiliations:** ^1^ Department of Radiotherapy The First Affiliated Hospital of Zhengzhou University Zhengzhou Henan People's Republic of China; ^2^ Department of Blood Purification The First Affiliated Hospital and Institute of Nephrology, Zhengzhou University Zhengzhou Henan People's Republic of China

To the Editor

1

Extranodal natural killer/T‐cell lymphoma, nasal type (ENKL) is an invasive non‐Hodgkin's lymphoma originating from mature NK cells or NK‐like T cells.^1^ The clinical manifestation of ENKL is atypical and highly variable, depending on the location of the disease and its histology. Various clinics are exploring the efficacies of different regimens such as radiotherapy,^2^ chemotherapy,^3^ chemoradiotherapy,^4^ autologous hematopoietic stem cell transplantation (AHSCT),^5^ allo‐HSCT,^6^ and other new drugs, but no optimal regimen has yet been reported for ENKL treatment. This study retrospectively analyzed and summarized the clinical data of 269 cases of ENKL treated in our hospital from 2007 to 2017. The goal of the study was to compare the effects of various treatment regimens on the survival of patients and gather evidence and experience in the individualized clinical treatment of ENKL.

Patients were evaluated weekly during treatment and followed up after treatment according to the institutional policy.^7^ Clinical examinations, imaging assessments, and pathological examinations were employed to evaluate the treatment response at every cycle and one month after the end of treatment according to the adapted Cheson's standard criteria.^8^ While complete response (CR) was defined as no evidence of residual disease, partial response (PR) was defined as a reduction of at least 50% of the pretreatment tumor burden. Stable disease (SD) was defined as less than 50% residual tumor burden or no disease progression, while progressive disease (PD) was characterized by an increase of greater than or equal to 20% in the maximal diameter of the tumor burden or appearance of new lesions. The overall response rate (ORR) was calculated as CR + PR. Treatment‐related toxicity was evaluated based on the National Cancer Institute's Common Toxicity Criteria (version 3).^9^ Progression‐free survival (PFS) was calculated from the date of diagnosis to the date of identification of disease progression and was censored at the date of the last follow‐up visit. Overall survival (OS) was calculated from the date of diagnosis to the date of death from any cause and was censored at the date of the last follow‐up visit. OS and PFS rates were analyzed using the Kaplan–Meier method.

As a result, a total of 269 patients (175 men and 94 women) fit the diagnostic criteria^10^ for ENKL. The median age of the patients was 44 years (range: 10–78). However, 27.1% of the patients were under the age of 30, indicating a decrease in the age of onset of ENKL. Most cases were positive for cytoplasmic CD3 (89.5%), CD56 (85.1%), T cell‐restricted intracellular antigen 1 (81.0%), and granzyme B (78.1%), which were indicative of tumor cells originating from NK cells. While 72.1% of the cases were positive for the Epstein–Barr encoding region in situ hybridization (EBER‐ISH), 93.5% of them were positive for plasma Ki‐67, which was suggestive of highly malignant cells.

In stage I/II disease (Table 1), the CR rate and ORR were 89.7% and 93.1%, respectively, in patients receiving the DDGP + IMRT treatment (n = 29); 33.3% and 50.0%, respectively, in patients receiving the SMILE treatment (n = 6); 66.7% and 73.3%, respectively, in patients receiving the VIPD treatment (n = 15); 84.2% and 89.4%, respectively, in patients receiving the VIPD + IMRT treatment (n = 19); and 11.1% and 22.2%, respectively, in patients receiving the AHSCT treatment (n = 9). In stage III/IV disease, the CR rate and ORR in patients receiving the DDGP + IMRT treatment (n = 13) were 76.9% and 84.6%, respectively; while in patients receiving the SMILE treatment (n = 18), they were 77.8% and 88.9%, respectively; in patients receiving the VIPD treatment (n = 5), they were 20.0% and 40.0%, respectively; and in patients receiving the AHSCT treatment (n = 9) were 70.0% and 80.0%, respectively.

**Table 1:** Treatment modalities and responses of extranodal natural killer/T‐cell lymphoma, nasal type (ENKL) patients

	Regimens	Number of Patients	CR	PR	SD	PD	ORR (%)
Stage I/II	IMRT	11	5	0	6	0	45.5
	DDGP	57	47	4	2	4	89.5
	DDGP+IMRT	29	26	1	0	2	93.1
	SMILE	6	2	1	2	1	50.0
	VIPD	15	10	1	1	3	73.3
	VIPD+IMRT	19	16	1	1	1	89.4
	AHSCT	9	1	1	3	4	22.2
Stage III/IV	DDGP	72	52	7	0	13	81.9
	DDGP+IMRT	13	10	1	0	2	84.6
	SMILE	18	14	2	1	1	88.9
	VIPD	5	1	1	2	1	40.0
	AHSCT	10	7	1	1	1	80.0

Abbreviations: AHSCT, autologous hematopoietic stem cell transplantation; CR, complete response; DDGP, dexamethasone/cisplatin/gemcitabline/pegaspargase; IMRT, intensity modulated radiation therapy; ORR, overall response rate; PD, progressive disease; PR, partial response; SD, stable disease; SMILE, dexamethasone/methotrexate/ifosfamide/L‐asparaginase/etoposide; VIPD, etoposide/ifosfamide/cisplatin/dexamethasone.

In addition, for the whole cohort, the CR/ORR were 85.7%/90.5% in patients receiving the DDGP + IMRT treatment, 76.7%/85.3% in patients receiving the DDGP treatment, 66.7%/79.2% in patients receiving the SMILE treatment, 55.0%/65.0% in patients receiving the VIPD treatment, and 42.1%/52.6% in patients receiving the AHSCT treatment.

At a median follow‐up of 56 months (range: 1–120), five patients had died; three of hemophagocytic syndrome (HPS) and two of HPS and multiple organ dysfunction syndromes (MODS). In patients with stage I/II disease (Figure 1), the 1, 2, and 3‐year OS and PFS rates were 86.7%/85.2%, 79.3%/75.2%, and 62.9%/60.3%, respectively, for the DDGP + IMRT regimen; 82.0%/80.2%, 78.4%/76.5%, and 63.1%/60.5%, respectively, for the DDGP regimen; and 56.3%/52.1%, 41.2%/39.8%, and 38.1%/36.2%, respectively, for the SMILE regimen. The 1‐year OS and PFS rates for the AHSCT regimen were 38.3% and 35.2%, respectively. The 1‐year OS and PFS rates for the VIPD regimen were 78.9% and 76.3%, respectively. In patients with stage III/IV disease, the 1, 2, and 3‐year OS and PFS rates were 73.2%/72.1%, 43.9%/40.2%, and 32.7%/29.6%, respectively; for the SMILE regimen, 71.7%/69.9%, 38.7%/36.9%, and 28.5%/26.8%, respectively; for the DDGP + IMRT regimen; and 69.9%/64.3%, 36.3%/34.9%, and 25.6%/22.3%, respectively, for the DDGP regimen. The 1‐year OS and PFS rates for the AHSCT regimen were 66.5% and 65.1%, respectively. The 1‐year OS and PFS rates for the VIPD regimen were 51.3% and 49.2%, respectively.

**Figure 1:**
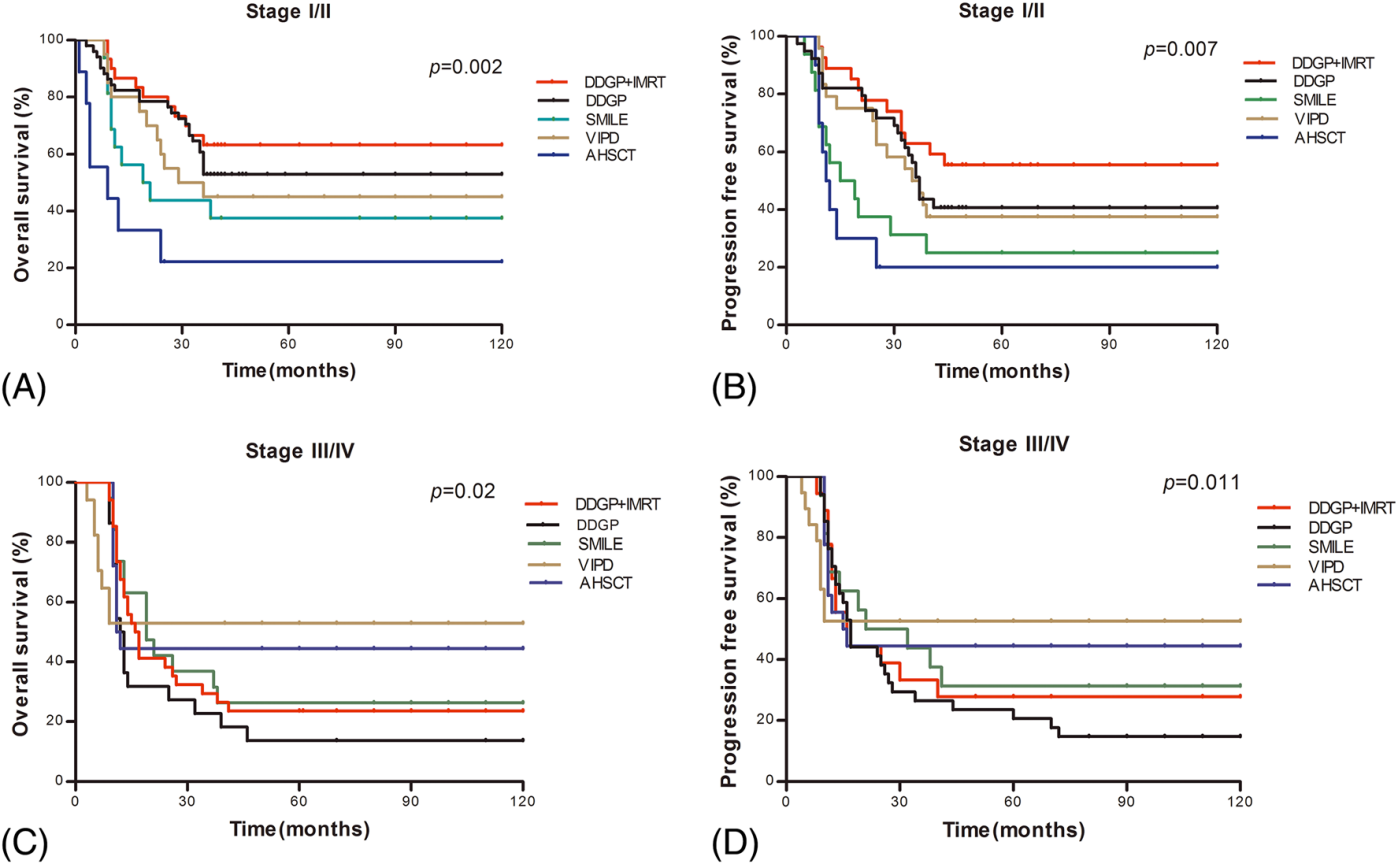
Kaplan‐Meier survival curves for all patients with extranodal natural killer/T‐cell lymphoma, nasal type (ENKL). A, The 1, 2, and 3‐year overall survival (OS) rates in patients with stage I/II disease. (.002). B, The 1, 2, and 3‐year progression‐free survival (PFS) rates in patients with stage I/II disease. (.007). C, The 1, 2, and 3‐year OS rates in patients with stage III/IV disease. (.02). D, The 1, 2, and 3‐year PFS rates in patients with stage III/IV disease. (.011) [Colour figure can be viewed at wileyonlinelibrary.com]

Besides, for the whole cohort, the 1, 2, and 3‐year OS and PFS rates were 79.2%/77.6%, 59.1%/56.1%, and 45.7%/43.6%, respectively, for the DDGP + IMRT regimen; 75.9%/72.3%, 57.4%/55.7%, and 44.3%/41.4%, respectively, for the DDGP regimen; and 64.7%/62.3%, 42.6%/40.1%, and 35.4%/32.9%, respectively, for the SMILE regimen. The 1‐year OS and PFS rates for the VIPD regimen were 65.1% and 62.8%, respectively. The 1‐year OS and PFS rates for the AHSCT regimen were 52.4% and 50.2%, respectively.

Our analysis shows that the DDGP + IMRT regimen results in significantly better outcomes in patients with ENKL. For stage I/II patients who cannot tolerate radiotherapy, the DDGP regimen is a better option, while for stage III/IV patients, the SMILE regimen is more effective.

## CONFLICT OF INTEREST

The authors declare that they have no conflict of interest.

## AUTHOR CONTRIBUTIONS

Daoke Yang and Zhangsuo Liu designed the research and edited the manuscript, Chunzhao Yang analyzed and interpreted the data, and drafted the paper. Yingjuan Zheng, Ping Wang, Tiansong Liang collected data and provided patient specimens.

## ETHICAL APPROVAL AND CONSENT TO PARTICIPATE

The study was conducted in accordance with the Declaration of Helsinki, International Conference on Harmonization guidelines and relevant laws and regulations. Approval for this observational study was obtained from the Medical Ethics Review Committee from The First Affiliated Hospital of Zhengzhou University.

## CONSENT FOR PUBLICATION

Not applicable.
